# Postural stability during illusory self-motion—interactions of vision and touch

**DOI:** 10.1007/s00221-025-07100-0

**Published:** 2025-06-10

**Authors:** Yingjia Yu, Avijit Bakshi, Alexander S. Panic, Paul DiZio, James R. Lackner

**Affiliations:** Ashton Graybiel Spatial Orientation Laboratory, MS 033, Brandeis University, 415 South Street, Waltham, MA 02454 USA

**Keywords:** Illusory body motion and displacement, Balance in virtual reality, Touch in virtual reality, Spatial disorientation, Balance during motion illusions

## Abstract

The role of vision in stabilizing balance has long been recognized, and previous studies have shown that non-supportive fingertip touch can enhance postural stability. However, the interaction between haptic feedback and the illusion of self-motion remains underexplored. We investigated how different phases of visual motion (no motion, visual motion, self-rotation and displacement illusion), motion order (stationary first vs. motion first), and fingertip cutaneous feedback jointly influence balance and the dynamics of haptic contact. Using a head-mounted display, we presented a virtual room that rotated around the standing participants’ vertical axis. Participants viewing the rotating scene soon experience illusory self-motion and displacement. We examined how the moving visual scene destabilized posture and how it interacted with tactile cues that typically stabilize balance. Our findings revealed differential effects in classical and stochasticity-sensitive analyses. Postural regulation was distinctly influenced by motion phase, order, and tactile feedback. Changes in motion perception—no motion, visual motion, and apparent self-rotation—were linked to both classical and stochastic aspects of postural sway. In contrast, motion order specifically influenced balance metrics encoding stochasticity, with no effect on those filtering out stochastic variability. Notably, the influence of past visual motion perception persisted, affecting postural sway even after motion ceased. The stabilizing effects of touch were reaffirmed, and motion perception significantly influenced the applied touch forces. Both stochastic and non-stochastic attributes of balance and touch force are responsive to visual motion perturbations and illusions, though motion order exclusively affects stochastic dynamics. These findings provide insights into multisensory interactions.

## Introduction

Human balance control relies on sensory inputs to activate muscular responses that adjust forces on the base of support (BOS), thereby regulating center of pressure (CoP) excursions. Visual, vestibular, auditory, proprioceptive, and somatosensory inputs, along with reflex mechanisms, work together to enable maintenance of an upright posture.

Visual elicitation of self-motion influences balance. Motion perception arises from either external object movement (afferent) or self-motion, with optic flow playing a key role in sensing self-motion during movement through extra-personal space (Gibson 1950). While the central visual field dominates object motion perception, the peripheral field is particularly sensitive to environmental motion and strongly affects self-motion and displacement direction perception, as shown by studies on optical flow (Gibson 1950; Dichgans and Brandt 1978; Horiuchi et al. 2017).

By distinguishing self-motion from external motion, vision—working in coordination with the vestibular system—enhances postural stability through optic flow, retinal slip, extraocular muscle afferents, and visual fixations (Chaudhary et al. 2022). Retinal slip not only induces optokinetic eye movements but also provides feedback to reduce sway, aided by oculomotor signals like vergence (Kapoula and Le 2006). While both retinal slip and extraocular detection via motor commands or muscle afferents contribute to stability during large body sway, extraocular afferents dominate in smaller sway scenarios (Guerraz and Bronstein 2008). Peripheral vision, which senses environmental motion aids quiet stance and reduces postural sway in the direction of visual stimuli (Berencsi et al. 2005). Guerraz and Bronstein (2008) suggest that the "peripheral dominance" in visual stabilization is likely due to the size of the stimulated field rather than a specific functional specialization of peripheral vision for postural control. Supporting this idea is the finding that the retinal periphery is sensitive to radially structured optical expansion when it conveys information about imminent collision (Stoffregen and Riccio 1990), while remaining insensitive to radial optic flow specifying postural sway (Stoffregen 1986).

Dichgans and Brandt revolutionized the understanding of visual influences on postural control through their pioneering studies on “vection,” the sensation of self-motion induced by moving visual scenes. Their seminal review sparked extensive research, revealing considerable individual variability in vection strength (Dichgans and Brandt 1978). Studies comparing postural sway under eyes-closed and eyes-open conditions with vection strength induced by various types of visual flow (e.g., oscillatory, smooth) demonstrated a strong predictive relationship between vection intensity and sway differences (Apthorp et al. 2014; Apthorp and Palmisano 2014; Palmisano et al. 2014a, b). These findings also highlighted the limitations of traditional linear measures of postural sway in capturing its full complexity.

Non-visual sensory channels, including vestibular, auditory, and tactile systems, contribute to detecting physical self-motion, inducing illusory self-motion, and stabilizing postural sway. The vestibular system, in coordination with the visual system, is essential for distinguishing self-motion from external motion. It provides six-dimensional estimates of physical self-motion—three axes of translation and three axes of rotation—through the otolith organs and semicircular canals thereby supporting postural stability (Cullen 2019).

Motion perception following rotational vestibular stimulation can evoke sensations of either self-motion or environmental motion (Kolev 2019). In postural research, illusory self-motion is typically generated using optic flow, though rotating auditory stimulation can produce similar effects (Lackner 1977). Tactile stimulation of the soles of the feet, can also induce illusory self-motion and displacement (Lackner and DiZio 2013). Integration of motion cues from vestibular and somatosensory systems facilitates the perception of object motion, self-motion, and overall postural control (Takamuku et al. 2024). Static auditory cues have been shown to reduce postural sway in both congenitally blind and sighted individuals tested with eyes closed (Easton et al. 1998). Similarly, non-mechanically supportive fingertip contact with a stationary surface significantly decreases postural sway, even under challenging conditions such as tandem Romberg stance (Holden et al. 1994; Jeka and Lackner 1994).

In touch stabilization studies, participants typically stand on a force plate that measures ground reaction forces and calculates the medial–lateral (ML) and anterior–posterior (AP) center of foot pressure (CoP). Changes in fingertip contact forces precede adjustments in the CoP by approximately 300 ms, highlighting the rapid and effective role of tactile feedback in stabilizing posture (Jeka and Lackner 1994). Remarkably, this “light touch” strategy is more effective than vision in reducing sway. Participants naturally adopt an average fingertip contact force of about 40 g, but studies have shown that forces as low as 5 g can attenuate postural sway (Lackner et al. 2001).

Individuals with vestibular loss lose balance within seconds when standing heel-to-toe with their eyes closed. However, when allowed light fingertip contact with a stable surface, these individuals demonstrate greater stability than normal subjects without touch under the same conditions in the dark (Lackner et al. 1999). This finding highlights that light touch cues can be even more effective than vestibular input in reducing postural sway.

Virtual reality (VR) enables the visual induction of self-motion and displacement through immersive environments. Modern head-mounted displays (HMDs) with wide fields of view (FOV) and head-tracking capabilities have proven valuable for studying the impact of visual motion on balance performance (Soltani 2019; Soltani and Andrade 2021). These devices effectively block external visual input, enhancing the sense of immersion. When standing subjects are immersed in a rotating virtual environment with a vertical axis of rotation, they experience an illusion of self-rotation (vection) around their yaw axis after a brief delay. Vection becomes more pronounced when realistic visual stimuli are used; for instance, naturalistic 3D or textured room displays evoke vection more quickly, intensely, and consistently than patterns like polka dots or alternating stripes (Wann and Rushton 1994; Schulte-Pelkum et al. 2003; Richards et al. 2004; Riecke and Schulte-Pelkum 2006).

Stabilogram Diffusion Function (SDF) Analysis. SDF is a quantitative method used to study postural control and assess the dynamics of human standing balance. It characterizes the stochastic characteristics of CoP fluctuations, elucidating the varied temporal-scale structure of sway, and enabling insights into the underlying control mechanisms of postural stability. Introduced to postural control by Collins and De Luca (1993), it is based on the idea that CoP movements resemble a random-walk process influenced by neuromuscular control mechanisms. Analyzing how these movements “diffuse” over timescales helps to differentiate between short-term and long-term postural control strategies.

A SDF computes the mean squared displacement (MSD) of CoP over increasing time intervals (τ), analyzing its diffusion characteristics to identify different regimes of postural control. The value of τ varies between the smallest time unit (1/fs) and typically a few seconds (2–5 s). For a given τ window width, the window is slid along the data sequence to compute the average MSD. Slope changes in a Log–Log Plot Analysis of MSD vs. sliding temporal window width reveal two primary control regimes: short- and long-term control. Short-term (open-loop control, automatic) is characterized by a smaller diffusion coefficient, where CoP fluctuations resemble a random walk (diffusive behavior) mainly dictated by intrinsic biomechanical noise. Long-term (closed-loop feedback-based control) CoP movements are more constrained, characterized by active neural corrective feedback mechanisms that help maintain balance.

The SDF is characterized by a set of parameters, including for example, the Hurst Exponent, Diffusion Coefficient, Critical Timescale, and Area Under the Curve (Chiari et al. 2002). Some parameters, like Diffusion Coefficient, can be computed for both short-term and long-term timescales. In the short term, it represents spontaneous postural sway-induced CoP fluctuations without significant feedback corrections (related to exploratory behavior or neuromuscular noise). Higher values suggest instability due to weak neuromuscular control. In the long term, it represents active balance control, reflecting the effectiveness of sensory feedback in stabilizing posture (Mitra and Fraizer 2004). A higher Diffusion value suggests poor control or compensatory instability. The Critical Timescale Interval is the transition point between short- and long-term regions, indicating the time scale at which the postural control system shifts from passive to active control (Hufschmidt et al. 1980). The Scaling Exponent reflects how CoP movements scale over time, and deviations from expected values indicate abnormal balance control strategies (Collins and De Luca 1995; Duarte and Zatsiorsky 2000).

Traditional versus SDF analysis offer different types of insights about postural control. The former (e.g., average fluctuations of CoP position and velocity amplitudes, sway area) are static metrics and reveal overall magnitude or spread of postural sway over a duration of time. SDF, by contrast, shows how the system controls balance over varied timescales, not just duration passed. Some of the inferences provided by SDF, which are unamenable by traditional analysis are—how sway diffuses over increasing time intervals, short-term (open-loop) and long-term (closed-loop) control strategies, how past movements influence future movements, underlying control mechanisms—sensory feedback or neuromuscular noise—and at what timescales feedback control kicks in, parametric diagnosis of specific neurophysiological processes—e.g., exploratory behavior, anticipatory vs. reactive control, or feedback delay. Other advantages of SDF over traditional averages are that it provides a model-free characterization by not relying on assumptions about the sway being linear or Gaussian and captures both stochastic and deterministic aspects of balance (Doyle et al. 2008). In studies of quiet stance sway, SDF is more sensitive than traditional CoP metrics as it can detect subtle differences in postural control that can be missed by traditional metrics, for example in early-stage neurological disorders or under different sensory manipulations (e.g., vision, touch, surface type), where the characteristics of postural control mechanisms can be more nuanced.

The present study measured the dynamics of multisensory integration in balance control, focusing on how vision and touch interact to maintain balance during conditions of visually induced apparent self-rotation and displacement (SR&D) and the presence of fingertip contact with a physical stationary surface and its removal. It explored whether vision would dominate, causing the fingertip contacted surface to be perceived as moving with the subject, or if it would result in the loss of the self-motion illusion. Also addressed was whether touch, which usually stabilizes posture, would be altered by illusory self-motion. The goal was to identify specific postural stability markers that might reflect the influences of perceived environmental motion versus self-motion, and any persisting effects on balance after the self-motion illusion had subsided.

We examined the effects of visual motion phase and order, along with touch, on two types of balance metrics:

1. *Traditional metrics*: Average fluctuations in the fore-aft (AP) and lateral (ML) directions of body sway.
2. *Metrics sensitive to stochastic correlation changes*: Stabilogram Diffusion Functions (SDF) and associated parameters, which capture balancing dynamics across different time scales.

The visual motion scenarios were divided into the following phases:

1. *Stationary scene (SS)*: A stationary virtual environment.
2. *Visual rotating scene (VRS)*: A virtual environment in motion, but no perceptual illusion of self-rotation.
3. *Self-rotation & displacement (SR&D)*^1^: The perceptual illusion of 360° self-rotation and displacement, while the visual environment is perceived as stationary.

## Hypotheses and objectives

1. *Effects of visual motion on balance metrics*: Based on previous studies, we hypothesized that visual motion would affect balance metrics across both traditional and SDF-based measures. Specifically, we anticipated that onset of Self-Rotation & Displacement (SR&D) would increase postural sway more than initial exposure to Visual Rotating Scene (VRS) prior to SR&D, and VRS would increase sway more than Stationary Scene (SS). Regarding directional effects, we hypothesized that VRS would influence both fore-aft (AP) and lateral (ML) sway, but we were uncertain whether SR&D would affect both directions equally.
2. *Interaction between vision and fingertip touch*: We sought to determine whether visual motion would dominate sensory integration, making the fingertip-touched surface feel as though it moved with the subject, or whether it would make the subject feel stationary, when the touch contact was made. Based on prior literature suggesting that touch stabilizes posture, we hypothesized that fingertip touch would reduce postural sway fluctuations induced by visual motion. Specifically, we expected touch to mitigate sway in both the AP and ML directions during visual rotation (SR&D). However, we were agnostic about potential interactions between fingertip touch and different visual motion conditions.
3. *Magnitude of fingertip touch forces*: We aimed to determine whether the magnitude of fingertip touch forces would vary across different phases of visual motion. Specifically, we examined whether illusory self-rotation (SR&D) would disrupt fingertip touch control. We expected the normal forces to hover around 40 g, which aligns with the most sensitive range of the cutaneous receptors of the fingers. However, we had no a priori hypothesis regarding changes in the magnitude or direction of forces in response to different motion phases.
4. *Effects of motion order on balance metrics*: We hypothesized that the order of visual motion conditions—stationary first, followed by visual rotation, versus rotation first, followed by stationary—would differentially affect fluctuations in balance metrics. However, we were uncertain whether these effects would manifest in both balance metrics, simple average postural sway fluctuations vs. SDFs encoding stochastic changes.

## Materials and methods

### Apparatus and measures

There were four main measuring devices used:

*Foot force plate.* An AMTI (Advanced Mechanical Technology, Inc.) dual force plate (Model 0392) was used to monitor the pressure exerted by each foot against time allowing us to compute medial–lateral (CoP_ML_) and anterior–posterior center of pressure (CoP_AP_). The weight fractions (WF) under the feet were computed from the vertical force under each foot. The sampling frequency was 200 Hz.

*Touch force plate.* A miniature AMTI force plate (model ZMCE 65) was used to detect contact forces of the right index finger in touch trials sampled at 200 Hz. The touch contact plate had a smooth square metal surface (225 cm^2^) attached horizontally to a vertical metal stand, parallel and lateral to the subject’s sagittal plane and approximately at the subjects’ waist height. The finger-tip touch force magnitude was recorded from the touch plate and its CoP was computed.

**Fig. 1:**
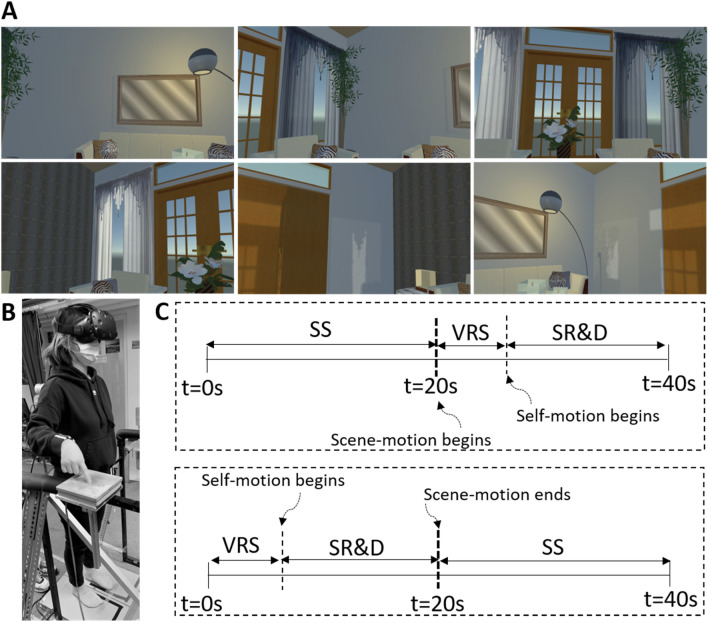
**A** Aspects of the complex visual environment that subjects saw in 3D and color in the HMD. **B** A subject wearing the HMD is shown standing on a force plate, touching the finger touch plate. **C** The three visual motion phases and two motion order transitions at *t* = 20 s. Stationary to moving (top panel) and moving to stationary (bottom panel). *SS* stationary scene; *VRS* visual rotating scene; *SR&D* self-rotation and displacement

*Head mounted display.* An HTC Vive™ Head Mounted Display (HMD), ~ 600 g in weight, rendering a VR scene at a resolution of 1080 × 1200 pixels per eye and a refresh rate of 90 Hz, was worn by individual subjects throughout the experiment. Its binocular display provided a 110-degree horizontal and vertical field of view. The HMD presented a well-structured virtual environment simulating a “living room”, with four-walls, ceiling, and floor and inner dimensions of width, depth, and height = 4.7, 4.7, and 3 m. The room was built with *Unity™* software, and its interior was highly textured, with doors, windows, sofa, tea table, plant in a pot, and other interior decorations (wall painting, flower vase, etc.). Figure 1A shows six snapshots of the scene as seen by subjects “standing” in the center of the room. The subject's field of view displayed the room image, with the only variation being whether the image remained stationary or was in motion. In every 40 s trial, the room was stationary half of the time, and the other half it was rotating at 60 deg/s. Depending on trial type, the room would be stationary or rotating at the start of a trial. The axis of room rotation coincided with the Z-axis of the standing subject.

*Camera.* An iPhone camera was used during each trial to capture the progression of time displayed by the force plate control software on a computer screen, along with the subject's verbal reports of self-motion. These recordings provided a synchronized view of the experimental timeline and the subject's responses. Following the trials, the experimenter reviewed the recordings to extract the precise timestamps corresponding to the subject’s verbal reports of induced self-motion and displacement. This ensured accurate synchronization of subjective reports with the collected experimental data.

*Subjects.* Fourteen subjects were recruited from Brandeis students and staff, their ages ranged from 18–55 years, 8 females and 6 males, one left-handed, all in good health. In pre-screening, all showed low susceptibility to motion sickness during HMD motion exposure. All subjects signed an informed consent form.

*Conditions.* There were 24 trials each lasting 40 s, divided into four sets of six: 12 of the trials started with 20 s of room rotation, followed by 20 s of the room stationary. The other 12 trials had the opposite order. Two independent variables were tested in the experiment:

- Independent variable 1: no touch or touch of the right index finger.
- Independent variable 2: start with no motion, followed by visual motion, or start with visual motion, followed by no motion.

In total, there were four different conditions, all counterbalanced across subjects. Direction of rotation of the virtual room was also counterbalanced.

*Procedure.* Subjects were familiarized with the phenomenon of self-motion and displacement before experimental trials by experiencing it firsthand. While seated in a chair wearing the HMD, they viewed the virtual room rotating and verbally reported when they began to sense combined SR&D. During the experimental trials, subjects stood quietly in stocking feet, with their feet parallel and aligned to guide tapes on the dual foot force plates. Foot positions were checked before each trial to ensure proper alignment. Subjects were informed of the upcoming trial condition prior to its start. In no-touch conditions, subjects kept their hands relaxed by their sides. In touch conditions, they used their right index finger to contact a touch force plate, centered approximately 35 cm forward and rightward from their midline. There was no rendition of the touch force plate visible within the VR environment. Before each trial began, subjects were instructed to close their eyes. When ready, they opened their eyes and simultaneously said “open.” The experimenter then started data collection^2^ and confirmed by responding, “collecting.” If room rotation occurred, either at the trial's start or mid-trial, subjects reported "moving" as soon as they experienced SR&D. This verbal response was recorded and synchronized with the collected data. For safety, a second experimenter acted as a “spotter,” ready to assist the subject in case of balance loss. Once the trial concluded, the experimenter instructed the subject to relax and close their eyes. Post-trial, subjects rated their dizziness, sleepiness, and fatigue levels on a 0–10 Likert scale, where 0 represented their state upon entering the laboratory and 10 represented the worst they had ever felt. If a subject's rating reached 3, the experiment was paused until their rating returned to 1 or 0. To minimize fatigue and allow for adjustments to the HMD's rotation direction, a 5-min rest break was scheduled after every 12 trials. The total experimental session lasted approximately one hour. Figure 1B illustrates a typical subject performing a touch trial.

### Data processing and analysis

*Timestamp synchronization.* A camera simultaneously recorded subjects' verbal reports of “moving” via the audio channel, while the visual channel captured the force plate data collection temporal progress bar displayed by custom-built software on a computer monitor. The experimenter later timestamped the exact moment of the “moving” utterance with ± 5 ms accuracy.

*Filtering:* The CoP and the raw force components data from both force plates were filtered by a 5th-order 5 Hz lowpass Butterworth filter (the *butter* function in MATLAB).

For each force plate, *CoP in AP and ML* coordinates was computed from

$$Co{P}_{AP L or R } = \frac{-{M}_{ML}}{ {F}_{Z L}} + {X}_{AP} \quad C{oP}_{ML L or R } = \frac{{M}_{AP}}{{F}_{Z R}} + {X}_{ML}$$

where F_Z_ represents the normal component of the ground reaction forces under the subjects’ feet and L and R subscripts stand for the Left and Right feet. M represents the moment or torque along either AP or ML directions. X represents position offset with the origin centered at the middle between the two feet.

*Weight fraction (WF):* The WF was determined from the vertical component (Z) of the force (F) measured in the left and right plates using the following equations:

$${WF}_{L}= \frac{{F}_{Z L}}{{F}_{Z L} + {F}_{Z R}} \quad {WF}_{R}= \frac{{F}_{Z R}}{{F}_{Z L} + {F}_{Z R}}$$

*Components of the net center of pressure (CoP*_*net*_*)*: This was computed from the left and right WF and CoP_L_ and CoP_R_ derived above:

$$Co{P}_{net} = {WF}_{L}*C{oP}_{L} +{WF}_{R} *C{oP}_{R}$$

The AP and ML components of this CoP_net_ are referred to as CoP_AP_ and CoP_ML_ in the “Results” section.

*Touch forces* Finger touch force magnitudes in Z-axis (F_Z_) and horizontal axes (F_AP_ and F_ML_) were measured directly from the touch plate readout.

*Fluctuations* The means of the absolute values of the fluctuations were computed for CoP_AP_, CoP_ML_, WF, and touch force fluctuations, for each trial.

*Stabilogram diffusion functions (SDF) analysis:* Quiet stance has deterministic and stochastic components that are amenable to treatment by SDF analysis. We used the methodology introduced by Collins and DeLuca (1993, 1995) to analyze our data. The mean squared displacement (MSD) of sway fluctuations is calculated for different time intervals, $\tau$= *t*_2_ − *t*_1_, using the equation *MSD*(*t*_2_ − *t*_1_) = <[*q*(*t*_2_) − *q*(*t*_1_)]^2^>, where q represents a time-series, as a function of time t, of fluctuations about the mean of the variables: (i) the components of the CoP*:* CoP_AP_ and CoP_ML_, (ii) the weight fraction, WF, and (iii) the finger touch forces. SDFs have the form of a power function with the SDF value (C) proportional to the time interval between comparisons raised to an exponent, given by $C=D{\tau }^{\alpha }$, where D is the diffusion constant and $\alpha$ is the exponent of the power law, which is also referred as the Hurst Exponent: E_H_ = α/2, and τ is a sliding window of different timescales across which the MSD is computed. The critical size of the temporal-scale window where the exponent E_H_ = 0.5 is referred to as the Critical Time (T_C_). At E_H_ = 0.5, the motion is Brownian; at E_H_ < 0.5, motion is antipersistent and negatively correlates with the future; at E_H_ > 0.5 the motion is persistent and the past correlates positively with the future. The Critical Time T_C_ represents the temporal divide between persistent and antipersistent behavior.

### Dependent variables and statistical analyses

For statistical comparisons between the conditions, dependent variables derived from the fluctuations of WF and the AP and ML components of CoP were analyzed in a 3-way MANOVA, within subject design, 3 (Motion phase) × 2 (Touch and No Touch) × 2 (Order). The three levels of the motion phase factor were: (i) stationary scene (SS), (ii) visual rotating scene (VRS) but no SR&D, and (iii) SR&D; the two levels of order of the scene presentation were “start with no motion followed by motion” or “start with motion followed by no motion”, as shown in Fig. 1C top and bottom panel, respectively.

From the SDF analysis of fluctuations of CoP_AP_, CoP_ML_ and WF, following the equation $C=D{\tau }^{\alpha }$, four dependent variables were derived for each. These derived SDF parameters were (i) the total area under each of the traces for different motion phases (AUC), (ii) the Hurst exponent (E_H_) satisfying, $\alpha =2{E}_{H}$, determined by the slope of log(C) plotted against $\text{log}\left(\tau \right)$, (iii) the short-timescale diffusion coefficient (D) which measures the stochastic activity on average, and (iv) the critical time point (T_C_) at which the power $\alpha =2{E}_{H} :=1$. Both 2-way and 3-way interactions among the independent factors were analyzed for the four DVs. For pairwise comparisons, we computed the contrast (type: repeated) between different levels of factors. Multivariate analyses incorporated Pillai’s Trace for significance at the 0.05 level.

## Results

### Raw sample data

**Fig. 2:**
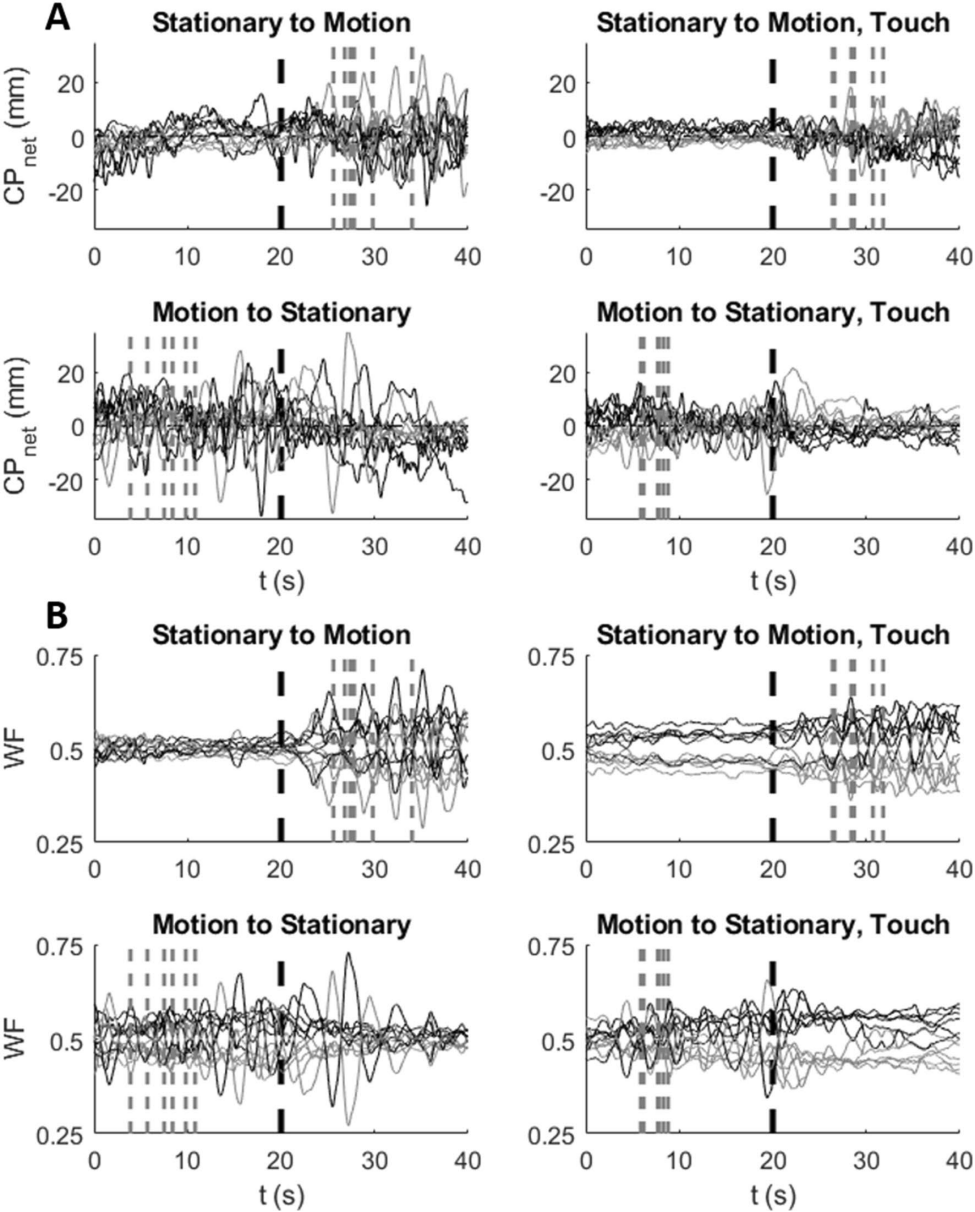
Sample data for a typical subject. The bold black vertical dashed line at *t* = 20 marks the change from stationary to visual motion (top panel) in the HMD or vice versa (bottom). The gray vertical dashed lines mark the onset times of the reported illusion of SR&D for the six trials of each condition. **A** CoP_net_ traces across the six trials for each of the four conditions (by motion order and touch). The AP component is in black and ML in gray. **B** WF traces for the right (in black) and left (gray) foot. As the sum of WFs always equals one, the traces fluctuate symmetrically about the 50% horizontal level

Figure 2A, B provide raw sample data of net CoP and (WF), respectively, for a typical subject’s six trials for each of the four conditions. The left four panels in A and B are without fingertip touch, the right four are with touch. The top two in A and B are for the stationary to motion transition, the bottom two are the reverse.

### Classical analysis with mean fluctuation magnitudes

**Fig. 3:**
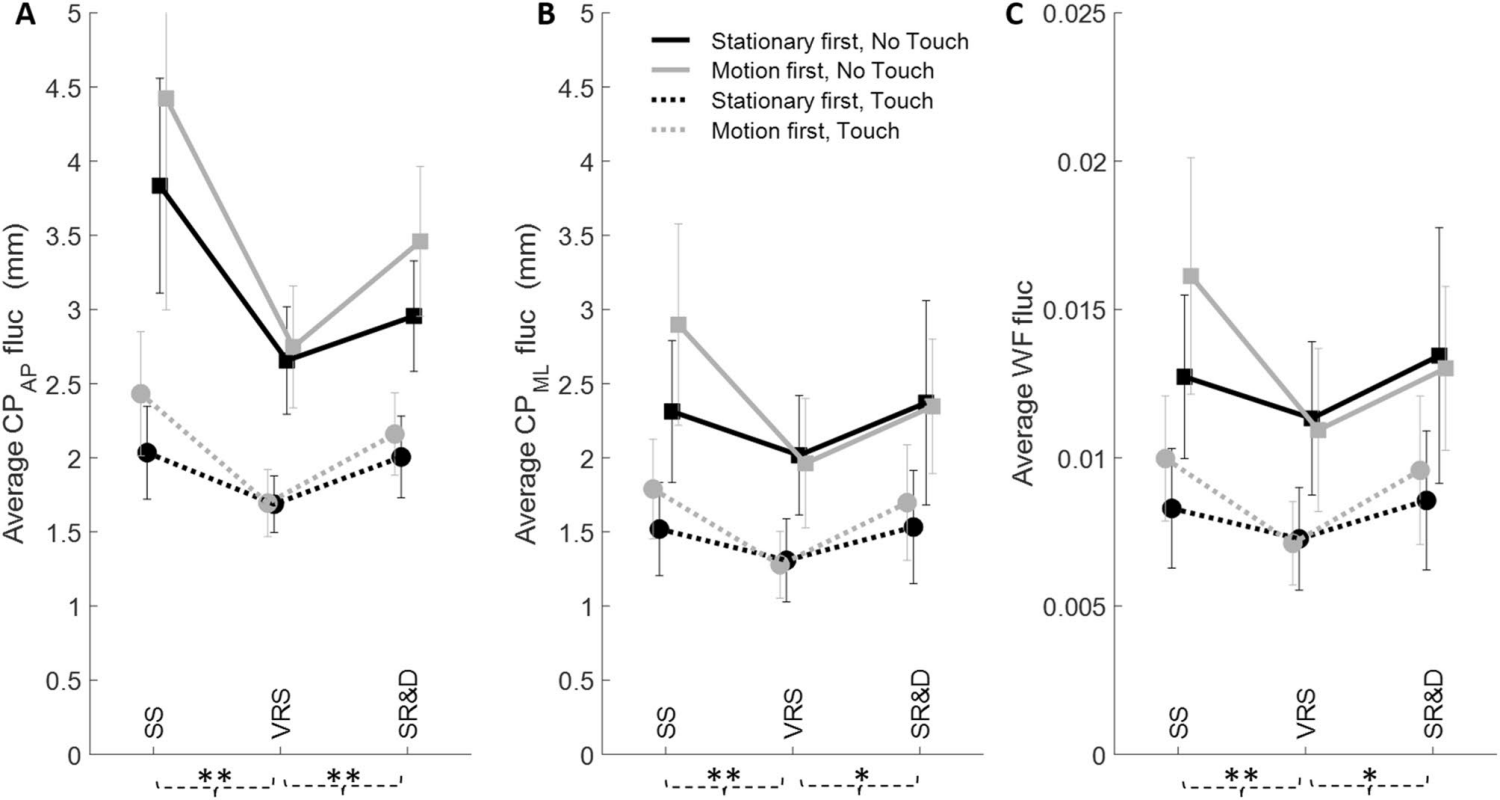
Mean fluctuation magnitudes and standard deviations of CoP_AP_ (**A**), CoP_ML_ (**B**) and WF (**C**) across the three motion phases and for the four distinct conditions. Black lines are for the order Stationary to Motion; gray for Motion to Stationary. Solid lines are no-touch, while dashed lines are touch. *SS* stationary scene; *VRS* visual rotating scene; *SR&D* self-rotation and displacement

Table 1A summarizes the statistical results for the classical metrics, the mean absolute fluctuations of CoP_AP_, CoP_ML_, and WF. Significances for both MANOVA and ANOVA at the *p* < 0.05 level (Pillai’s trace) are reported in black (grayed when univariate was significant but corresponding multivariate was not; we do not interpret these unless we have an a priori prediction). Effect sizes η^2^ are stated within parenthesis. Figure 3A–C show the average CoP_AP_, CoP_ML_ and WF^3^ fluctuation magnitudes (with 1 SD), respectively. In the AP direction, the perception of SR&D consistently led to higher mean net CoP fluctuations (> 3 mm) than during VRS (< 3 mm). Motion phase showed significant main effects for all the above three fluctuation variables (refer Table 1A). Planned comparisons showed significant differences in fluctuations, for all three, during environment rotation (VRS) versus stationary (SS) and also for illusion of SR&D versus VRS. Touch significantly stabilized balance, reducing the CoP and WF fluctuations by almost half. There was no order effect: the stationary to motion condition versus the motion to stationary condition did not differ in average balance measure fluctuations. These findings corroborate the significant effects of visual motion, and SR&D, on balance and emphasize the stabilizing effect of touch on all motion-type phases and all motion order conditions.

**Table 1:** Summary of multivariate, univariate, and paired comparison tests for the Motion Phase (3) × Motion Order (2) × Touch (2) design on postural metrics. Only significant p-values are reported, with effect sizes in parentheses. (A) Mean fluctuations in CoP_AP_, CoP_ML_ and WF. (B) Parameters derived from the SDF of the three fluctuations

	Independent variables			Interactions		MP paired comparisons		
		Motion phase (MP) (3)	Motion order (MO) (2)	Touch (T) (2)	MP * T	MP * MO	SS vs. VRS	VRS vs. SR&D

(A) Mean Fluc								
MANOVA		< 0.05 (0.22)		< 0.001 (0.85)				
ANOVA DVs	Fluc CP_AP_	< 0.01 (0.35)		< 0.01 (0.78)	< 0.04 (0.23)		< 0.01 (0.43)	< 0.01 (0.49)
	Fluc CP_ML_	< 0.04 (0.22)		< 0.01 (0.80)			< 0.01 (0.44)	< 0.03 (0.32)
	Fluc WF	< 0.05 (0.21)		< 0.01 (0.79)			< 0.01 (0.43)	< 0.04 (0.31)

(B) SDF Fluc								
MANOVA		< 0.001 (0.70)	0.006 (0.99)	< 0.02 (0.99)		< 0.05 (0.60)		
ANOVA DVs	CP_AP_ area			< 0.01 (0.43)				
	CP_AP_ hurst exponent	< 0.001 (0.64)					< 0.001 (0.76)	= 0.04 (0.29)
	CP_AP_ critical point	< 0.02 (0.27)				< 0.01 (0.39)	< 0.04 (0.29)	
	CP_AP_ diffusion coeff		= 0.05 (0.26)	= 0.001 (0.57)				
	CP_ML_ area			< 0.01 (0.52)				
	CP_ML_ hurst exponent	< 0.001 (0.61)					< 0.001 (0.72)	
	CP_ML_ critical point	< 0.01 (0.32)				< 0.01 (0.53)	< 0.03 (0.33)	
	CP_ML_ diffusion coeff		< 0.01 (0.48)	< 0.001 (0.62)				
	WF area			< 0.01 (0.48)				
	WF hurst exponent	< 0.001 (0.64)					< 0.001 (0.75)	
	WF critical point	< 0.01 (0.37)				< 0.01 (0.49)	< 0.01 (0.45)	
	WF diffusion coeff		< 0.01 (0.45)	= 0.001 (0.58)

### SDFs of fluctuations

We tested whether the SDF was sensitive to (i) perception of apparent SR&D, (ii) to touch, and (iii) the order of transitioning from stationary to visual motion or vice versa. Mean squared displacements were computed for different temporal-scale windows for CoP and WF fluctuations and touch forces.

**Fig. 4:**
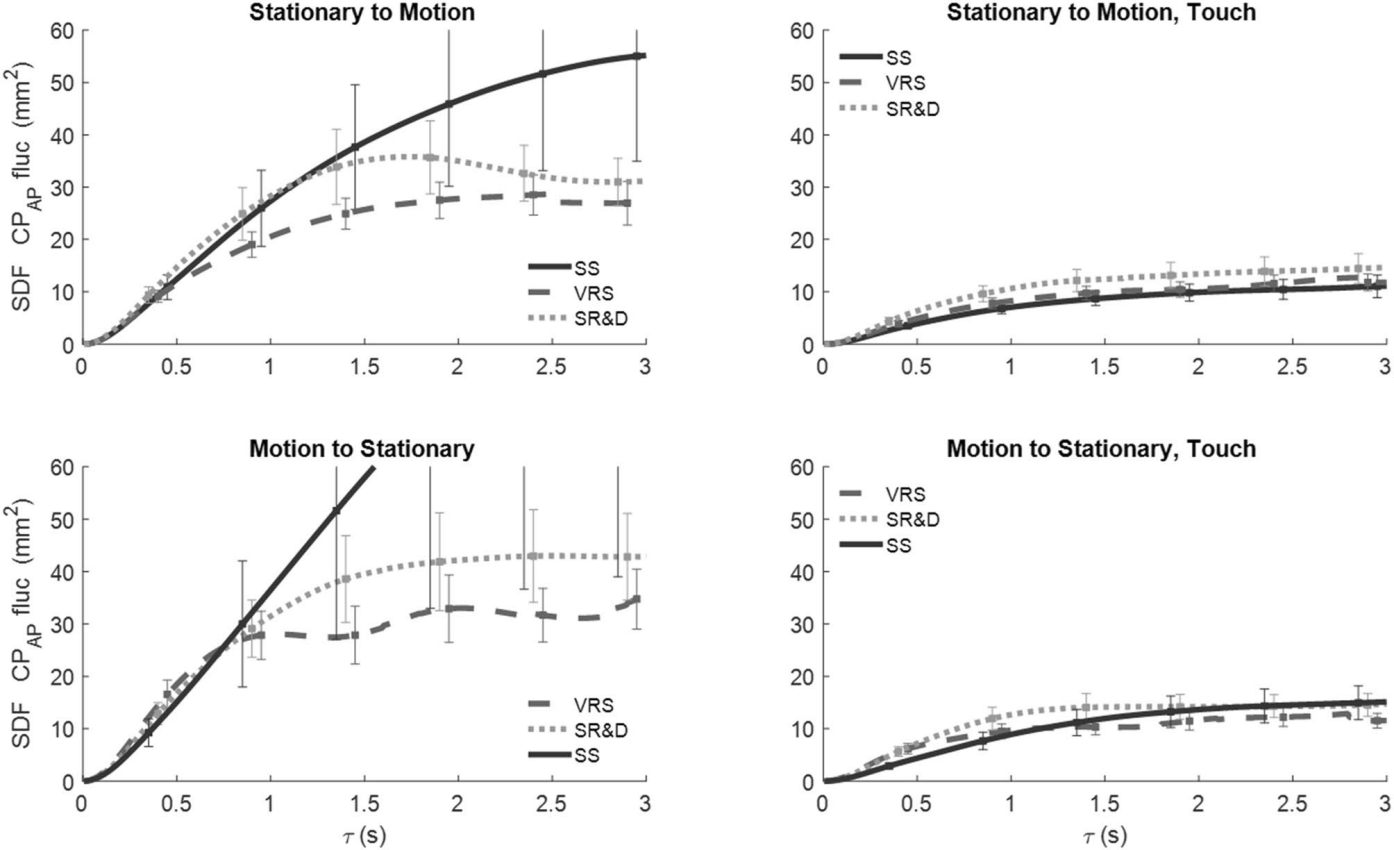
SDF vs. temporal-scale width of the CoP_AP_ fluctuations for all touch, motion-order conditions, and the three motion phases

*SDF of CoP*_*AP*_ fluctuations against the temporal window width (τ) for the three motion-phase epochs—SS (solid black line), VRS (dashed line), perceived SR&D (dotted line) are shown in Fig. 4. Each trace is the average across all the subjects, and the error bars represent the standard errors. Four distinct patterns emerge from the average SDF traces. For the no-touch conditions (left panels), the SDF trace of CoP_AP_ fluctuations are greater: (1) during SS than all other motion phases in the longer time scale (solid line above dashed or dotted lines), (2) during SR&D than for VRS (dotted higher than dashed), (3) for SS when preceded by motion (solid trace in the bottom-left panel) than when not (solid black line in top-left). (4) The SDF traces of CoP_AP_ fluctuations in the touch conditions (right panels) are smaller than those in no-touch conditions (left panels), and are virtually comparable across all touch conditions.

**Fig. 5:**
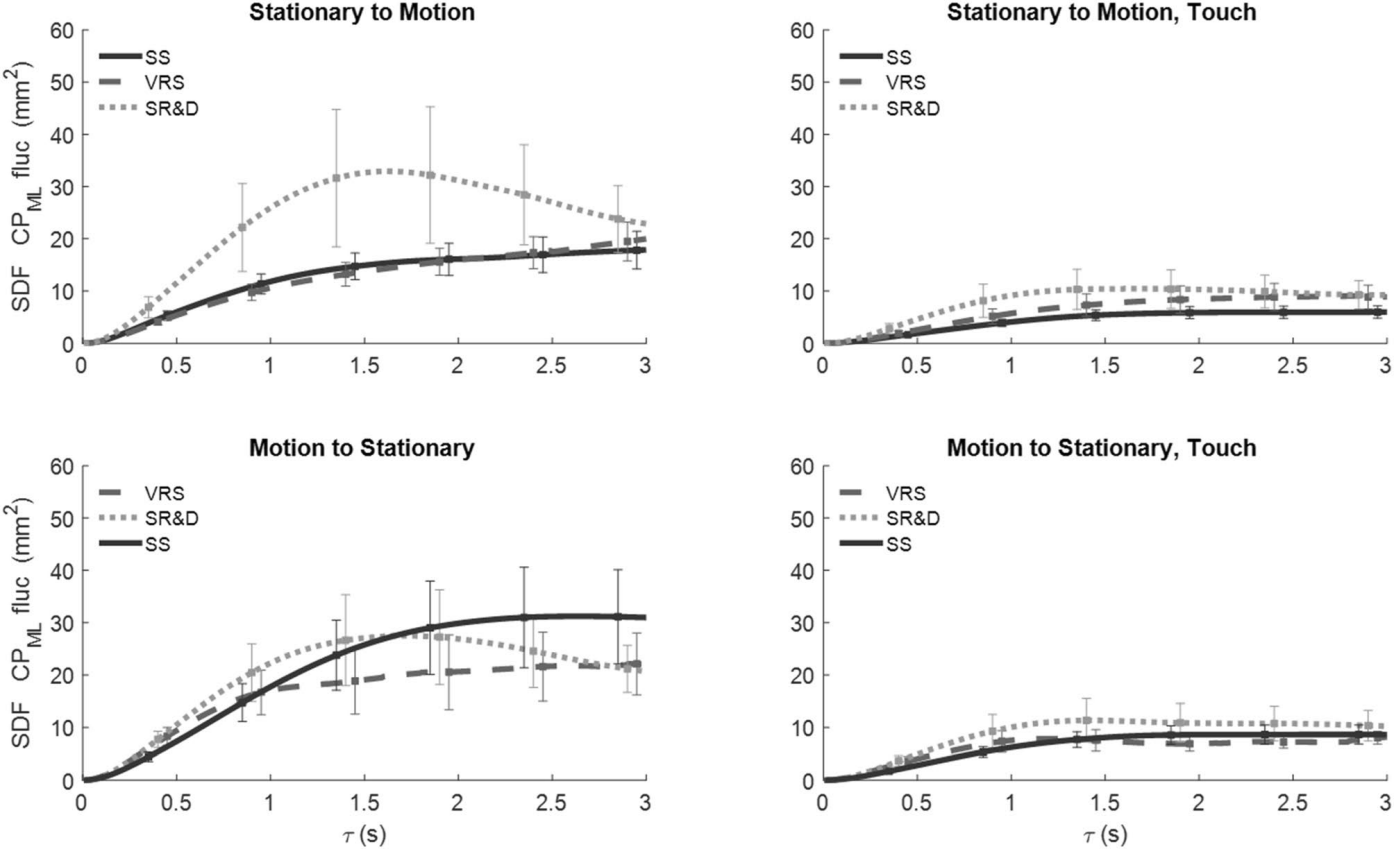
SDF vs. temporal-scale width of the CoP_ML_ fluctuations for all touch and motion-order conditions, and the three motion-phases

*SDF of CoP*_*ML*_ fluctuations against τ are shown in Fig. 5, where all line indicators and placement of condition plots are the same as in Fig. 4. In contrast to the CoP_AP_ fluctuations SDF, when a trial started with SS, CoP_ML_ fluctuations were higher during SR&D compared to both SS or VRS (dotted line higher than others in top-left panel), but there was no difference between SS and VRS phases (solid and dashed lines in top-left). When the trial started with motion, CoP_ML_ traces changed for different motion perceptions (bottom-left). The touch condition (right panels) SDFs were lower than no-touch (left panels) on average; furthermore, with touch the three SDFs are not different from one another, just as was the case for the CoP_AP_ results, demonstrating that light touch reduces both CoP_ML_ and CoP_AP_ fluctuations significantly regardless of motion phase.

**Fig. 6:**
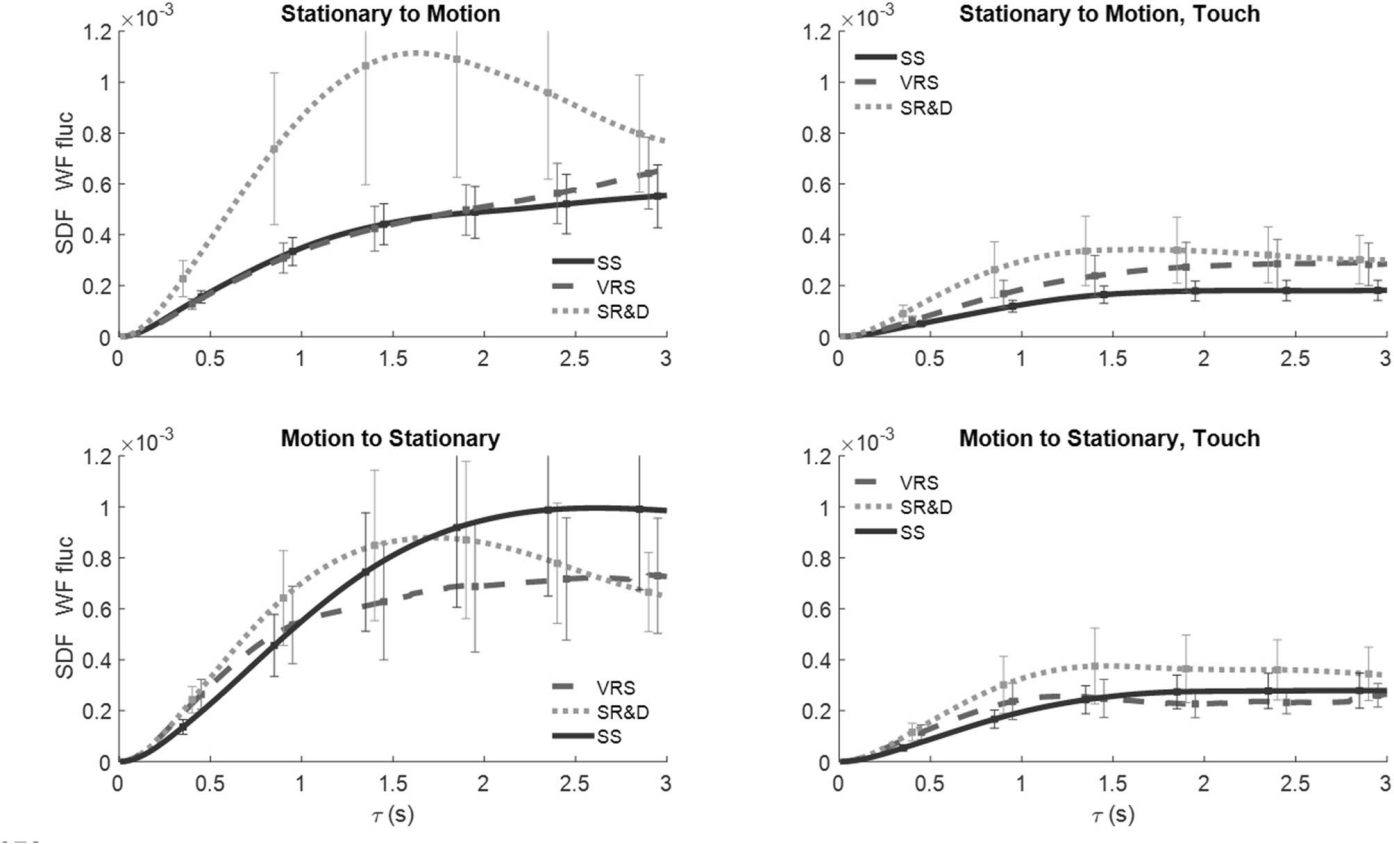
SDF of WF fluctuations

*SDF traces of WF* fluctuations of the left foot^2^ are plotted against τ in Fig. 6 in the same format as Figs. 4 and 5. Similar to CoP, WF fluctuations for touch conditions (right panels) were reduced significantly vis-a-vis no-touch conditions (left panels). In no-touch trials, when VRS followed the SS, the WF fluctuations during SR&D (gray dotted trace in top left) were significantly higher than those in SS and VRS phases. However, when the trials started with VRS (bottom left panel) the traces of each of the three phases are closer together. Touch greatly attenuates the WF fluctuations across all comparisons.

Four DVs, defined in the methods, were extracted from the SDF traces: Area, Diffusion Coefficient (D), Critical Timescale (T_C_), and Hurst Exponent (E_H_). A three-factor MANOVA, with a 3 (Motion-phase: SS, VRS, SR&D) × 2 (No-touch and touch) × 2 (Order of display: stationary first or moving first) design, was performed on SDFs of the AP and ML components of CoP fluctuations and on the WF fluctuations^2^. Table 1B presents the statistical results for the dependent variables derived from the SDF of these fluctuations. Broadly, these measures derived from the SDFs of each of the three fluctuation measures (of CoP_AP_, CoP_ML_, and WF) showed significant main effects for touch, order and motion-phase. The E_H_, T_C_ and D parameters provide a more detailed characterization of stochasticity and the presence of persistent or anti-persistent phenomena. For future comparisons, Table 2 includes a summary of descriptive statistics for these three measures, presented as mean (standard error).

**Table 2:** Descriptive statistics, including means and standard errors (in parentheses), for the Hurst exponent (E_H_), Critical Time (T_C_), and Diffusion Coefficient (D) parameters derived from the SDF analysis of CoP_AP_, CoP_ML_, and WF fluctuations, presented across the independent factors of motion phase, order and touch

		Motion phase			Touch		Motion order	
		SS	VRS	SR&D	NT	T	SS 1st	VRS 1st
CP_AP_	E_H_	0.746 (0.004)	0.780 (0.004)	0.769 (0.005)	0.763 (0.003)	0.767 (0.005)	0.764 (0.004)	0.766 (0.004)
	T_C_	0.819 (0.073)	0.620 (0.043)	0.653 (0.050)	0.763 (0.045)	0.631 (0.045)	0.699 (0.040)	0.695 (0.049)
	D	8.554 (2.935)	8.005 (0.949)	8.790 (1.558)	12.518 (2.443)	4.382 (0.576)	7.258 (1.042)	9.642 (1.978)
CP_ML_	E_H_	0.751 (0.004)	0.782 (0.004)	0.772 (0.005)	0.769 (0.004)	0.768 (0.004)	0.768 (0.004)	0.769 (0.003)
	T_C_	0.886 (0.062)	0.768 (0.057)	0.748 (0.060)	0.795 (0.061)	0.807 (0.053)	0.805 (0.060)	0.797 (0.050)
	D	3.921 (0.680)	4.434 (0.904)	6.497 (2.029)	7.057 (1.580)	2.843 (0.698)	4.396 (1.008)	5.505 (1.264)
WF	E_H_	0.751 (0.004)	0.782 (0.004)	0.773 (0.005)	0.770 (0.004)	0.767 (0.004)	0.768 (0.004)	0.770 (0.003)
	T_C_	0.881 (0.059)	0.766 (0.066)	0.751 (0.063)	0.784 (0.061)	0.815 (0.061)	0.807 (0.063)	0.792 (0.058)
	D	1.2 (0.18)E-4	1.4 (0.2)E-4	2.1 (0.4)E-4	2.2 (0.28)E-4	9.1 (1.2)E-5	1.4 (0.24)E-4	1.7 (0.22)E-4

*Motion phase* showed significant multivariate main effects. For each of the three fluctuations the significant differences in motion phase arose from two DVs, Hurst exponent and Critical Timescale, but not for Area and Diffusion Coefficient (Table 1B). Pairwise comparisons of the motion-phase factors showed that stationary (SS) significantly different from environment rotating (VRS) for all three fluctuation measures (via both Exponent and Critical Timescale DVs). However, SR&D was significantly different from VRS only for AP fluctuations, via the Exponent DV only; none of the lateral fluctuations (CoP_ML_ and WF) showed any VRS vs SR&D differences.

*Touch* had significant multivariate main effects on SDFs of all three fluctuation measures; the common DVs sourcing these differences for all three measures, in the univariates, were Area and Diffusion coefficient. This result for touch stands in contrast to those for motion phase where all significances were originating from the other two DV pairs, Hurst exponent and Critical Point, and none from Area and Diffusion coefficient.

*Motion order* main effect was strongly observed on the lateral fluctuation measures but was only borderline significant in the foreaft AP fluctuation SDF. All order significances in the SDFs of lateral ML fluctuations sourced only from Diffusion-coefficient DV.

*Interactions*: There were significant two-way interactions between Motion-phase and Order in the SDFs of all three fluctuation measures—CoP_AP_, CoP_ML_, and WF—with all significant effects originating from a single dependent variable, the Critical Timescale. Motion-phase and Touch showed a significant interaction only at the univariate level for the fore-aft component (CoP_AP_) fluctuation, but not for the lateral components (CoP_ML_ and WF); however, as this effect was not significant at the multivariate level, therefore we do not interpret it. No significant interactions were found between Touch and Order, nor were there any three-way interactions in the SDF analysis.

These results indicate that (1) VRS and especially SR&D alters balance fluctuations, (2) non-supportive mechanical light touch significantly reduces fluctuations in all balance measures in all conditions, (3) prior exposure to motion increases the fluctuations during stationary conditions without touch, (4) the AP and ML components are distinctly influenced by phase and ordering of motion, for example, external environment motion versus self-motion affects fore-aft but not lateral components, while motion order effects lateral but not fore-aft, (5) effects of motion phase and touch can be encoded in distinct, mutually exclusive, balance metrics, for example, the motion phase influenced Hurst exponent and Critical Timescale, while Touch influenced Area under SDF and Diffusion coefficient, and (6) a phase and order interaction driven solely by T_C_ across all three fluctuation measures suggests that the temporal sequencing of perceived motion plays a crucial role in shaping the stochastic temporal structure of the stability dynamics.

### Touch forces

**Fig. 7:**
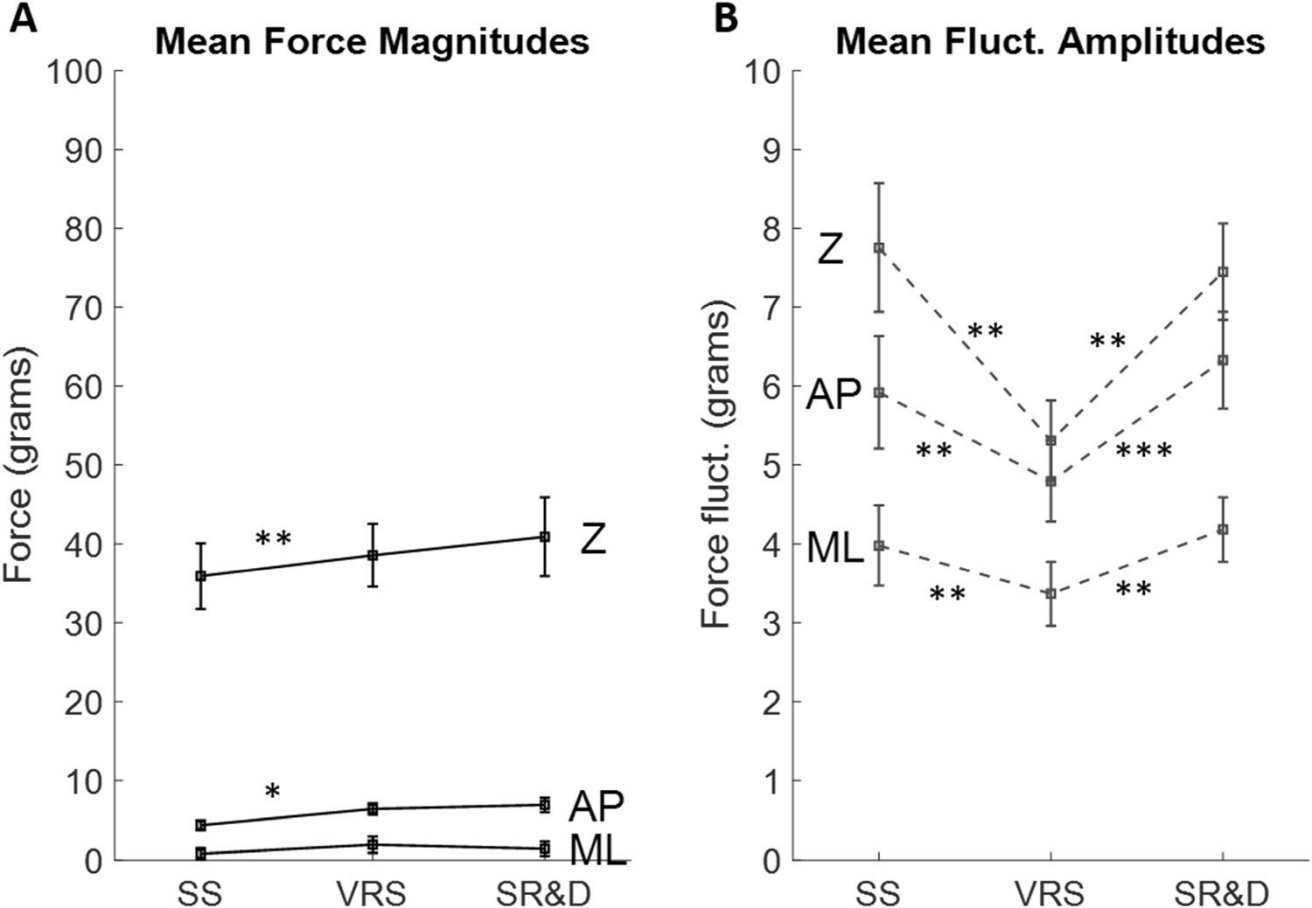
**A** The average force magnitudes along the three cardinal directions for each of the three motion phases. Remarkably, the normal Z component was tuned to maximal cutaneous sensitivity at 40 g for the illusory SR&D phase. **B** The average amplitudes of the fluctuations in the touch force. The amplitudes are smallest for VRS relative to both SR&D and SS across all directional components

The AP, ML, and Z finger touch forces were analyzed for the touch conditions and the significant results are summarized in Table 3A. There were significant average touch force magnitude differences with changes in motion state. Motion phase had significant effects on the normal touch force component (F_Z_) and the fore-aft component (F_AP_), but not on the lateral component (F_ML_). F_Z_ averaged 35.9 ± 4.2 g during SS, increased to 38.6 ± 4 g during VRS, and further to 40.9 ± 5 g during SR&D, as shown in Fig. 7A. Thus, touch forces during SR&D are tuned to the magnitude, 40 g, at which fingertip cutaneous receptors are known to exhibit peak sensitivity (Johansson and Westling 1987; Westling and Johansson 1987; Holden et al. 1994). The tangential components were much smaller in magnitude: average F_AP_ increased from 4.4 ± 0.6 g (SS), to 6.4 ± 0.7 (VRS), to 6.9 ± 0.9 (SR&D), while mean F_ML_ was between 0.5–2.5 g only. Motion state also significantly altered the average amplitudes of the fluctuations in each component of the applied touch force magnitudes, as seen in Fig. 7B. For AP they were 5.9 ± 0.7 (SS), 4.8 ± 0.5 (VRS) and 6.3 ± 0.6 (SR&D) grams; ML was slightly lower, 4 ± 0.5 (SS), 3.4 ± 0.4 (VRS) and 4.2 ± 0.4 (SR&D) grams; and Z slightly larger at 7.7 ± 0.8 (SS), 5.3 ± 0.5 (VRS) and 7.4 ± 0.6 (SR&D) grams. Thus, in comparison, exerted fingertip force fluctuations were lower during scene motion. This may indicate that environmental motion via optic flow or retinal slip enhances the CNS’ ability to stabilize balance, reducing the fluctuations in the force magnitudes, whereas illusory self-motion during the SR&D phase compromises this stabilization. The touch force fluctuation amplitudes are the only dependent measure we found that captures significant effects for both SS to VRS transitions and VRS to SR&D transitions.

**Table 3:** Summary of multivariate, univariate, and paired comparison tests for the Motion Phase (3) × Motion Order (2) design on touch force metrics (all touch trials). (A) *p*-Value (effect size) for the mean force magnitudes and their average fluctuations along the three cardinal directions. (B) Results on parameters derived from the SDF of fluctuations in the touch forces

		Independent variables		Interaction	MP paired comparisons	
		Motion phase (MP) (3)	Motion order (MO) (2)	MP * MO	SS vs. VRS	VRS vs. SR&D

(A) Touch forces						
MANOVA		< 0.001 (0.58)		< 0.01 (0.43)		
ANOVA DVs	F_AP_	< 0.02 (0.29)			< 0.04 (0.30)	
	F_ML_	0.06 (0.19)				
	F_Z_	< 0.01 (0.36)			= 0.01 (0.41)	
	Fluc F_AP_	< 0.001 (0.50)			< 0.01 (0.55)	< 0.001 (0.74)
	Fluc F_ML_	< 0.01 (0.31)			< 0.01 (0.44)	< 0.01 (0.54)
	Fluc F_Z_	< 0.001 (0.51)			= 0.001 (0.55)	< 0.01 (0.55)

(B) SDF Fluc						
MANOVA		0.011 (0.64)		0.049 (0.58)		
ANOVA DVs	F_AP_ area	0.005 (0.33)		0.023 (0.25)		0.001 (0.56)
	F_AP_ hurst exponent					
	F_AP_ critical point					
	F_AP_ diffusion coeff	< 0.001 (0.46)				< 0.001 (0.64)
	F_ML_ area			0.029 (0.24)		0.022 (0.34)
	F_ML_ hurst exponent		0.004 (0.47)	< 0.001 (0.50)		
	F_ML_ critical point		0.041 (0.28)	0.007 (0.32)		
	F_ML_ diffusion coeff					0.011 (0.41)
	F_Z_ area	0.004 (0.35)				0.004 (0.49)
	F_Z_ hurst exponent					
	F_Z_ critical point					
	F_Z_ diffusion coeff	0.059 (0.20)				0.001 (0.56)

Motion phase affected selective parameters of the SDF of the fluctuations of the force magnitudes (Area and Diffusion coefficient, but not the Exponent and Critical Timescale) and only along specific force directions (AP and normal components, but not ML), as summarized in Table 3B. Paired comparisons confirmed that the effects of different motion phases are encoded in distinct measures. Average touch force magnitudes are significantly altered with VRS relative to SS, but show no effect with onset of SR&D from ambient-motion (VRS). Differences between VRS and SR&D are captured only by the measures derived from the fluctuations – the average touch force fluctuation amplitudes and also by Area and Diffusion Coefficient parameters of the SDF of the fluctuations.

There were no significant touch force differences with changes in motion order. However, a significant interaction between motion phase and motion order was observed in both classical and SDF touch force analyses. In the classical analysis, this interaction was significant only at the multivariate level, suggesting that a linear combination of touch force magnitudes and fluctuation amplitudes contributed to the effect. In the SDF analysis, the interaction emerged selectively in the SDF parameters of the tangential touch force components, while not the normal component.

### Latencies of onset of SR&D

**Fig. 8:**
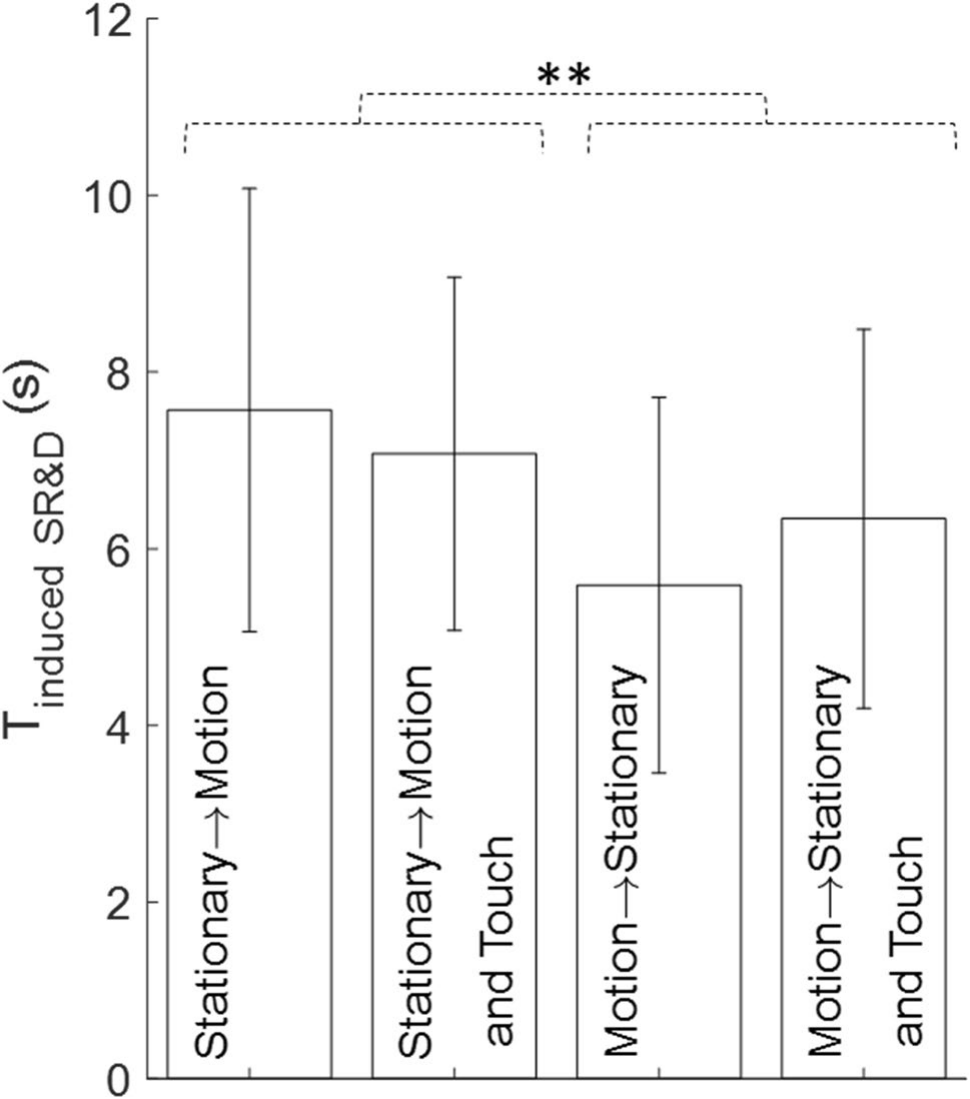
The temporal period for the induction of SR&D across the four conditions

The mean latencies of onset of SR&D, shown in Fig. 8, were 7.6 ± 2.5 s (stationary to motion without touch), 5.6 ± 2.1 s (motion to stationary without touch), 7.1 ± 2 s (stationary to motion with touch), and 6.3 ± 2.1 s (motion to stationary with touch). There was a significant effect of motion onset order (F(1,13) = 15.8, p = 0.002) and a significant touch and motion order interaction (F(1,13) = 5.8, p < 0.04). The average time of SR&D onset during the stationary to motion conditions (7.3 s) was significantly longer than the time of SR&D onset during the motion to stationary conditions (6 s).

## Discussion

### Visual motion affects posture

As discussed in the Introduction, it is well-established that visual motion induces changes in postural sway. Our task elicited illusory self-rotation and displacement (SR&D) using continuous 360° full-field virtual scene rotation of a well-structured room environment. Our findings revealed a distinction between environmental rotation (VRS) and illusory SR&D. In general, center of pressure (CoP) and weight force (WF) fluctuations were greater during SR&D than during the periods when only a moving visual scene was perceived. When the structured visual environment rotated in yaw, body sway was perturbed in all directions, as reflected by changes in the mean fluctuations and Stabilogram Diffusion Function (SDF) analysis. Furthermore, SR&D significantly increased postural sway compared to the sway observed during VRS. These effects were evident in specific parameters of the SDF, namely the Exponent and the Critical Timescale (Table 1B). 

Normally SR&D perception involves multisensory stimulation—optic flow, vestibular inputs that provide independent information about head movement, and proprioceptive and somatosensory information. A rotating visual environment in an HMD elicits conflicting sensory inputs over time, for example, when SR&D is perceived, there are no signals from the semicircular canals to indicate motion onset. One interpretation could be that the sensory conflict increases postural sway. Alternatively, Gibson (1966) argued that multisensory patterns can provide orientation information without requiring separate sensory inputs to compete. In our study, no change in vestibular semicircular canal or otolith organ input was triggered as the perception of self-motion was utterly illusory. Signals received by vision alone were strong enough to bring changes in postural balance, reflected by a noticeable increase in weight loading fluctuations and by the center of pressure fluctuations seen in the stochastic measures. When proprioceptive and somatosensory information were provided from the fingertip touch, they greatly reduced postural sway.

### Start with motion vs. start with no motion

Statistical analyses across all variables revealed a significant main effect dependent on the order of the motion phase (i.e., starting with a Stationary Scene transitioning to Scene Motion, or vice versa). When the task began with visual rotation and no touch cue was provided, the moving visual input destabilized postural control, resulting in a disruptive aftereffect once the scene transitioned to stationary. Interestingly, motion order did not affect the average CoP fluctuations in any direction. Its influence was exclusively observable in the SDF measures, which are sensitive to changes in stochasticity. Notably, the Diffusion coefficient, particularly in the lateral direction, emerged as the only parameter sensitive to motion order (Table 1B). During touch trials, motion order had no main effect on the touch force measures. However, a significant interaction effect between motion phase and motion order was observed, indicating that the stochastic aspects of postural balance and haptic touch characteristics varied with different motion perceptions, depending on whether the trial began with motion or ended with it.

Visual motion aftereffects might contribute to this effect. Andreeva (2016) found visual motion aftereffects of opposite sign could be induced by prolonged exposure to a moving environment. In our task, the sudden stop of scene motion might induce eye movements in the direction opposite to prior optokinetic nystagmus. This also could account for the slight discomfort, as reported by several subjects during stopping of the scene. In future studies, we will use eye tracking to measure how the eyes move during both the onset and stopping of visual motion.

### Touch vs. no touch

In all test conditions, non-supportive fingertip touch significantly reduced the mean fluctuations of the center of pressure (CoP) in both the AP and ML directions when visual motion was introduced (see Figs. 4 and 5). Notably, perceptual changes caused by moving visual input did not significantly interfere with the stabilizing effect of fingertip touch; touch consistently reduced postural sway across all conditions. Significant differences were also observed in the SDFs of CoP and weight (WF) fluctuations due to touch. This effect emerged in specific stochasticity-sensitive parameters, such as the Area of the SDF profile and the Diffusion Coefficient (Table 1B), but not in the Exponent or Critical Timescale. This contrasts with the effects of motion phase, which were observed in the latter pair but not in the former.

In our experimental design, the touch plate was always positioned to the right side of each subject, regardless of handedness. While this could be a limitation, it is mitigated by the fact that 13 of the total 14 subjects were right-handed. Out of the 14 subjects, 9 experienced the touch plate moving in coordination with their fingertip when contact was made, 1 subject felt the touch plate switch from not moving to moving with the fingertip, and 4 subjects did not perceive any touch plate motion. Regardless of the perception of touch plate motion, light touch consistently reduced postural sway. For the 10 subjects who experienced the touch plate moving with their finger, the motion of their fingertip and the touch plate were perfectly synchronized with their perceived body rotation, as though the plate were an extension of their own body.

Previous research (Bakshi et al. 2019) demonstrated that voluntary swaying in a slow rotating room (60°/s), was influenced by Coriolis perturbations, which significantly deviated the subjects' sway. This postural sway was also attenuated by active light touch and best described by stochastic measures. Similarly, postural sway induced by visual environment rotation in the present experiment followed a stochastic trajectory. SDF measures were instrumental in characterizing the changes in postural sway patterns induced by altered visual motion perception, as well as by the presence and absence of touch, and in understanding how these factors affect different aspects of postural stochasticity. The significant reduction in postural sway with fingertip light touch, observed in both actual and virtual rotation environments, underscores the role of touch in reducing the stochasticity of sway.

### Touch magnitude

When visual motion was introduced, the touch forces significantly varied across different motion phases in AP and Z directions (Table 3A). The average touch force magnitudes increased with motion, with the smallest values observed during the stationary scene (SS), intermediate values during the visual rotating scene (VRS), and the maximum forces during self-rotation and displacement (SR&D). Notably, the vertical touch force consistently hovered around 40 g during SR&D, while it was 5 g lower during the stationary phase (Fig. 7A). This aligns with previous research, which identified 40 g as the threshold for maximum cutaneous sensitivity in the fingers (Johansson and Westling 1987; Westling and Johansson 1987). Thus, under self-motion conditions, the central nervous system (CNS) appears to automatically tune into the optimal tactile sensory sensitivity. The average fluctuation amplitudes of the touch force also varied across different motion phases in all three cardinal directions. However, in contrast to the average force magnitudes, which were lowest during the SS phase, the fluctuation amplitudes were smallest during VRS, with both SS and SR&D exhibiting amplitudes significantly higher than those observed in VRS (Fig. 7B). We also observed a significant interaction between motion phase and motion order on the touch force measures.

Overall, these findings are consistent with touch belonging to an haptic long-loop cortical reflex that adjusts postural sway using somatosensory and proprioceptive information related to the activation of a complex network of areas, ranging from motion-specific areas to regions involved in visuo-vestibular integration, visual imagery, decision making, and introspection (Kovács et al. 2008). The modulation of balance through light touch contact is remarkably fast (250–300 ms), with EMG activity onset in the leg muscles controlling sway detectible at 150 ms and force generation to counter sway present 150 ms later, well below conscious reaction times (Jeka and Lackner 1995; Rabin et al. 2006).

### Interplay of visual motion perception, order and tactile feedback in postural stability

Touch generally stabilizes balance, but our findings suggest a complex interplay between visual perception and the tactile system in the acquisition of postural stability. Interactions between motion phase and motion order emerged in both classical and SDF analyses for touch force control (Table 3) but only in the SDF analysis for postural control (Table 1). The significant two-way interactions in the SDFs of all three fluctuation measures—CoP_AP_, CoP_ML_, and WF—highlight the systematic impact of motion sequencing on the stochastic attributes of postural stability. Notably, all significant effects were driven by a single dependent variable, the Critical-point (Table 1B), suggesting that the temporal-scale structure of stochastic fluctuations in balance dynamics is strongly influenced by the temporal sequence of different visual motion perceptions. The interaction between motion phase and motion order in the touch force analysis perhaps suggests that the sequential execution of motion phases has an integrated effect on force application, possibly reflecting adaptive strategies to maintain stability across varying movement conditions, but with lingering aftereffects. A possible explanation for this effect could be that when trials began with a stationary scene, vertical touch force fluctuations remained relatively unchanged across different motion phases; however, when trials started with scene motion followed by no motion, touch forces were greater when SR&D was perceived, and this effect persisted into the subsequent stationary phase. Interestingly, in the SDF analysis, the interaction between motion phase and order emerged selectively in the tangential touch force components but not in the normal component. The absence of an effect on the normal component suggests that vertical force may serve as an anchoring mechanism for balance maintenance, remaining stable within each balancing context and rapidly adapting to changes in visual perturbation, regardless of sequence. Future studies will further investigate whether lateral and fore-aft touch force adaptations are more sensitive to motion sequencing than vertical force adjustments. If confirmed, this would reinforce the idea that while tangential forces may play a key role in compensatory movements and fine adjustments to external visual perturbations, they do not directly govern balance stability, and therefore they may exhibit a hysteresis effect or latency in switching, which could explain why changes in motion order influence how subjects regulate forces applied along the horizontal plane. Understanding these dynamics could offer deeper insights into sensorimotor integration and the mechanisms underlying balance adaptation in varying sensory environments.

### Distinct effects across directions

The factors of motion phase, motion order, and touch exhibited unique effects along the three cardinal directions—fore-aft (AP), lateral (ML), and normal (Z). For instance, motion order had a stronger impact on the Diffusion Coefficient parameter of the SDFs of lateral fluctuations (i.e., CoP_ML_ and WF) compared to the fore-aft direction. In contrast, the distinction between SR&D and VRS in motion phase was only noticeable in the Exponent parameter of the fore-aft CoP fluctuation SDF, but not in the lateral direction. Motion phase significantly altered the average touch force magnitudes and fluctuation SDF parameters in the fore-aft and normal directions, but had no effect on the lateral ML direction. Interactions between the phase and order of motion were significant in the fore-aft and lateral touch force fluctuation SDF parameters, but not in the normal force component.

### SDF parameters capture distinct effects of motion phase, order and touch on balance

For each SDF trace, four dependent variables (DV) were derived: Area under the SDF curve (AUC), Diffusion Coefficient (D), Critical Timescale (T_C_), and Hurst Exponent (E_H_). The three independent factors—motion phase, order and touch—significantly influenced certain SDF parameters of CoP and touch force fluctuations, while others remained unaffected. For the CoP fluctuations, the motion phase factor significantly affects E_H_ and T_C_ but does not influence the AUC or D. The Hurst scaling exponent measures the self-similarity and memory of a time series, indicating persistence (E_H_ > 0.5), anti-persistence (E_H_ < 0.5) or random walk behavior (E_H_ ~ 0.5) of fluctuations over time. In this experiment scaling exponents were always found to be > 0.5 (see Table 2), meaning CoP movements exhibit persistence, where past movements are correlated with future movements in a consistent direction. Table 2 shows that the exponent of VRS > of SR&D > of SS, indicating that the vection illusion increased CoP fluctuation persistence, with the onset of visual motion amplifying this effect. Greater persistence implies a more pronounced drift-like component, which could stem from enhanced anticipatory control in maintaining balance. The Critical time (T_C_) represents the transition between short-term (open-loop) and long-term (closed-loop) control, marking the timescale when active postural control begins to dominate over passive control. A shorter T_C_ suggests a rapid response in postural control, whereas a longer T_C_ may indicate delayed corrective mechanisms. Table 2 shows that the two motion phases significantly reduced T_C_ compared to SS, implying that motion stimulus required faster neuromuscular corrections than stationary condition.

The second factor, touch, influences AUC and D but does not influence the complementary parameters E_H_ and T_C_. AUC represents the total extent of postural sway over different timescales, where a larger AUC suggests increased instability, while a smaller AUC indicates more controlled postural sway. Figures 5, 6, and 7 support this expectation, showing a significant reduction in AUC with touch.

The effect of touch on the Diffusion constant is especially important. In SDF analysis, D reflects spontaneous postural sway driven by biomechanical noise or exploratory behavior, where higher values suggest greater sway instability due to weak neuromuscular control. Table 2 shows about a four-fold reduction in D with touch, indicating a significant decrease in spontaneous postural sway-induced CoP fluctuations. The third factor, motion order, only affects D, with no effect on the other three parameters. In summary, motion phase affects the passive versus active aspects of balance control strategy, while touch and motion-order primarily influence the noise-dependent stochastic aspects of postural sway.

The SDF parameters of touch force fluctuations indicate: (i) The motion phase factor significantly changes AUC and D for fore-aft touch force fluctuations, has no effect on the lateral direction, and only affects AUC for the normal force component. (ii) The contrast between ambient motion versus self-motion phase is encoded only in AUC and D, with no influence on E_H_ or T_C_.

### Relevance for competing views of how posture and touch are stabilized

Our results have implications for understanding the basis for finger touch contact being able to stabilize balance. The prescient remarks of Gibson provide an important background: “To perceive is to be aware of the surfaces of the environment and oneself in it. The full awareness of surfaces includes their layout, their substances, their events, and their affordances. Note that this definition includes within perception, a part of memory, expectation., knowledge, and meaning -some part but not all of those mental processes in each case” (Gibson 1979, p. 255); and, “We are not accustomed to think of the hand as a sense organ since most of our day to day manipulation is performatory, not exploratory. …. The perceptual capacity of the hand goes unrecognized because we usually attend to its motor capacity….” (Gibson 1966, p. 123).

There have been several interpretations of how touch is related to stabilization of posture. Our original studies related the stabilization as being akin to a form of precision grip in which the finger and legs served as the two “pincers”. Given the short latencies of posture stabilization onset below conscious reaction times, we related the stabilization to the action of a long loop cortical reflex, also known as a transcortical reflex (Wiesendanger et al. 1975; Holden et al. 1994; Jeka and Lackner 1994). An alternative view has been that of functional integration or that posture stabilizes to afford stabilization of the hand contact with the surface. The functional integration hypothesis (or suprapostural task hypothesis) argues that posture is adaptively shaped to support the execution of supra-postural tasks and its modulation is subordinate to the demands of goal-directed behaviors (Riccio and Stoffregen 1988). Evidence for this hypothesis is found in postural sway attenuation and modulation in service of task performance during activities requiring fine motor control—such as visually guided movements, object balancing, manual aiming, and precision touching (Riley et al. 1999; Balasubramaniam et al. 2000; Stoffregen et al. 2000; Wulf et al. 2004; Haddad et al. 2013; Chen et al. 2015, 2018; Chen and Tsai 2015).

The functional integration hypothesis contends that such stabilization occurs only when task-specific precision is required. Riley et al. (1999), for example, found no sway reduction in a task demanding minimizing contact-point variability when subjects made contact with a pliable curtain. Other work has shown that rigid surfaces elicit stronger stabilizing effects than compliant ones (Franzén et al. 2011; Mauerberg-deCastro et al. 2014; Batistela et al. 2018; Moraes et al. 2018). A potential reason for diminished postural modulation when touching without a goal, or touching non-rigid contacts, could be that traditional sway metrics are overlooking subtle effects in more compliant contact conditions. This raises the question whether the effects of touching flexible surfaces could be aided by more sensitive measures—such as SDF analysis, which gives a detailed time course of control.

It is plausible that the postural control system flexibly integrates both sensory feedback and task constraints, with the relative influence of each depending on the specific haptic context. Several proposals have attempted to develop an integrated framework bringing competing hypotheses to a state of cooperative reconciliation, wherein each mechanism aptly fits in specific domains. For example, Mitra’s (2003) adaptive resource-sharing model suggests that under conditions of increased postural threat (e.g., reduced base of support, sensory challenges), sensory feedback is prioritized to maintain stability, and conversely, in stable configurations, the postural system allocates resources to support supra-postural goals when they are present. Chen and Tsai (2015) propose that the postural system operates as a flexible, context-sensitive mechanism even under safe, upright conditions.

We agree with the notion of supra-ordinate representations of postural control in touch stabilization experiments and in any tasks involving directed actions of the hands, arms, legs and whole body. Our view is that such control represents a physical necessity in order for the tasks to be carried out.

Previous work has dealt with how establishing finger contact with a surface can affect posture and the calibration of the arm. For example, when we reach and contact a target location on the surface, there is a brief period when the three-dimensional reaction force on the touching finger points at the shoulder of the reaching arm. This maintains the accuracy of arm directional calibration (Lackner and DiZio 2000). In touch of the finger to a surface, stabilization occurs in less than ≈ 150 ms, EMG activity is evoked in the muscles that will act to stabilize posture in another 150 ms. Even finger contact with a von Frey filament can, in subjects standing heel to toe, provide some stabilization at finger force levels of 5–10 g; by contrast, holding the finger above the force plate attempting to maintain it at an imagined location has no stabilization effect (Lackner et al. 2001). When a subject is standing heel to toe and the peroneus longus and brevis tendons of one leg are vibrated to elicit a tonic vibration reflex, the subject’s balance is greatly compromised. However, allowing the subject fingertip contact with a stable surface stabilizes the subject to the level of that seen with touch contact without vibration. Many subjects report that it feels as if the vibrator is not on (Lackner et al. 2000). The efficacy of touch stabilization has been shown for Parkinsonian patients as well. When such an individual has trouble in initiating locomotion, they can be unstuck if they lower their finger to touch a moving belt that “unsticks” them so that they can walk the full length of the moving belt (Rabin et al. 2015). Our studies are a testimony to Gibson’s wisdom that “The perceptual capacity of the hand goes unrecognized because we usually attend to its motor capacity…”. We are only beginning to glimpse the full range of his insights.

## Data Availability

No datasets were generated or analysed during the current study.

## Abbreviations

SS: Stationary scene
VRS: Visual rotating scene (no self-rotation illusion)
SR&D: Self-rotation and displacement (illusion)
WF: Weight fraction
SDF: Stabilogram diffusion function

## Footnotes

1 SR&D is similar to the classic circular vection (CV) illusion but carries a subtly distinct perceptual quality. CV typically refers to the sensation of self-rotation that would be elicited by rotating vertical stripes. In contrast, as detailed in the Methods section, our visual display featured a richly textured environment with varied objects and corners that, when viewed, participants report not only a sense of self-rotation (i.e., CV) but also a perceived 360-degree displacement with each full cycle of room rotation. To capture this meaningful addition to the perceptual experience, we refer to the phenomenon as SR&D rather than simply CV.

2 The latency between subjects reporting their eyes open and the triggering of data collection was negligible compared to the duration of each motion phase; thus, these delays were considered inconsequential for the purposes of this experiment.

3 Note that the mean fluctuations of WF for the left or right foot are equivalent but of opposite sign. Also, the SDF output for the left and right foot fluctuations are exactly equivalent because WF_left_ + WF_right_ = 1; = > Δ WF_left_ + Δ WF_right_ = 0; = >|Δ WF_left_ | =|Δ WF_right_|. So the left or right foot WFs gives the same results, because knowing one also determines the other.
